# Structural modelling and phylogenetic analyses of PgeIF4A2 (Eukaryotic translation initiation factor) from Pennisetum glaucum reveal signature motifs with a role in stress tolerance and development

**DOI:** 10.6026/97320630012416

**Published:** 2016-12-19

**Authors:** Aakrati Agarwal, Yashwanti Mudgil, Saurabh Pandey, Dhirendra Fartyal, Malireddy K Reddy

**Affiliations:** 1 Plant Molecular Biology Group, International Centre for Genetic Engineering and Biotechnology, Aruna Asaf Ali Marg, New Delhi-110067, India;; 2 Plant Molecular Biology Lab, Department of Botany, University of Delhi, New Delhi, India;

**Keywords:** eIF4A2, Eukaryotic initiation factor, helicase, structural modelling, Pennisetum glaucum

## Abstract

Eukaryotic translation initiation factor 4A (eIF4A) is an indispensable component of the translation machinery and also play a role in developmental processes and stress alleviation in plants and animals. Different eIF4A isoforms are present in the cytosol of the cell, namely, eIF4A1, eIF4A2, and eIF4A3 and their expression is tightly regulated in cap-dependent translation. We revealed the structural model of PgeIF4A2 protein using the crystal structure of Homo sapiens eIF4A3 (PDB ID: 2J0S) as template by Modeller 9.12. The resultant PgeIF4A2 model structure was refined by PROCHECK, ProSA, Verify3D and RMSD that showed the model structure is reliable with 77 % amino acid sequence identity with template. Investigation revealed two conserved signatures for ATP-dependent RNA Helicase DEAD-box conserved site (VLDEADEML) and RNA helicase DEAD-box type, Q-motif in sheet-turn-helix and α-helical region respectively. All these conserved motifs are responsible for response during developmental stages and stress tolerance in plants.

## Background

Pennisetum glaucum (L.) R. Br.) has not been extensively utilized for its genes providing abiotic stress tolerance including eukaryotic translation initiation factors (eIFs) that are involved in the alleviation of salinity and other environmental stresses in plants and animals. eIF4A2 is a member of the family of DEADbox RNA helicase which is the largest family of RNA helicases. The number of these proteins in Pennisetum glaucum is unknown. A soybean DEAD-box RNA helicase was found to be induced in presence of salinity stress [1]. A LOS4 RNA helicase was found to have activity in cold stress response of Arabidopsis [2].

Presence of at least a single isomer of eIF4A is mandatory for proper translation and mediation of response during abiotic and biotic stresses. An insight into the structure of PgeIF4A2 would give us a fair idea of its elements responsible for its role in various developmental and stress responses. Here, we use structural modelling technique that is widely employed, and aims at predicting the 3D structure of bio-molecules, relying largely on resources such as pattern/function and sequence. There is very few data regarding the structural modelling of RNA helicases in plants that are responsible for enhanced tolerance to abiotic stress. Knowledge of the (3D) structure of eIF4A2 from Pennisetum glaucum could pave way for better engineered crops tolerant to abiotic stress. However, 3D structure of eIF4A2 from Pennisetum glaucum is unknown till date. In the present study, we generate the 3D structure of eIF4A2 from Pennisetum glaucum based on the available human eIF4A3 (2J0S) structural homologue as template. The model structure PgeIF4A2 was validated with standard parameters (PROCHECK, PROSAII, Verify3D, RMSD). This study could prove useful in functional characterization of plant RNA helicases in response to plant stress tolerance. These particular domain sequences could be used with newer technologies like CRISPR to determine their roles in mediating both abiotic and biotic stress response mechanisms in plants.

## Methodology

### Sequence Analysis

Our lab has isolated the nucleotide sequence for PgeIF4A2 from Pennisetum glaucum. It was found to be over-expressed in a cDNA library under salinity stress and it was submitted to NCBI under the Accession Number EU856535.1 [3]. Structural modelling is initiated by looking in the Protein Data Bank for already known protein structures and the target sequence is used as the query [4] In this search, the PgeIF4A2 is compared with the sequence of each of the structures present in the database. Using PDB BLAST, the PgeIF4A2 was searched for a similar sequence [5] against the PDB. The BLAST results yielded X-ray structure of 2J0S, the eIF4A from Homo sapiens with 77 % similarity to PgeIF4A2.

### Modelling

Modeller 9.12 was used to generate the theoretical structure of PgeIF4A2 from P. glaucum. Protein structure prediction was done by comparative modelling. Various possible kinds of information about the target sequence were used by this program.

### Model validation of PgeIF4A2

#### Model evaluation

PROCHECK, ProSA-Web [6] and Verify 3d [7] were used to evaluate the model on the basis of geometrical and stereochemical constraints.

#### Root Mean Squared Deviation

In general terms, RMSD is used to represent the distance between two objects. In case of structural determination, this value is indicative of the degree of similarity between two three dimensional structures. Maximum similarity of structures is represented by a lower value. The RMSD value between the template 2J0S and PgeIF4A2 was calculated using MOE.

#### Phylogenetic analysis

Molecular Evolutionary Genetic Analysis (MEGA) software (version 4.0.02) [8], was used for carrying out the phylogenetic analysis of the sequences using N-J method with used bootstrap value 5,000 replicates.

## Results & Discussion

### Modelling of PgeIF4A2

**Figure 1:**
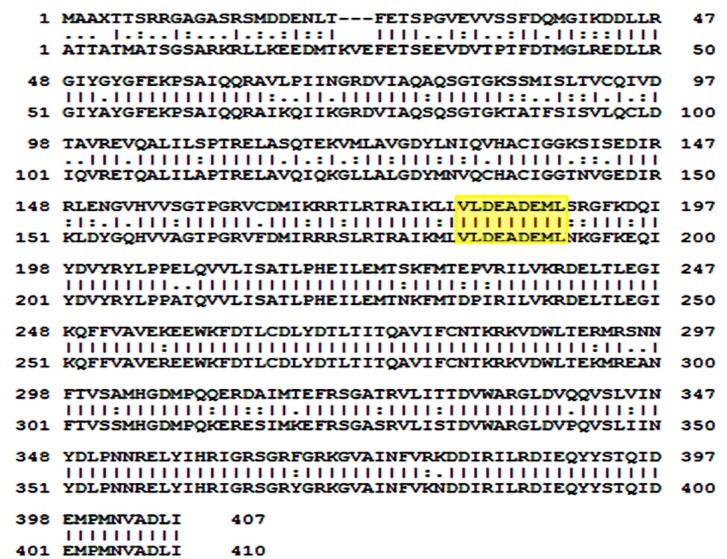
Alignment of deduced amino acid sequences of eukaryotic translation initiation factors from Homo sapiens (H. sapiens; template) and Pennisetum glaucum (P. glaucum; target) revealing the signature motifs for ATP-dependent RNA helicase and RNA helicase Q-motif binding employing pair-wise alignment tool.

**Figure 2:**
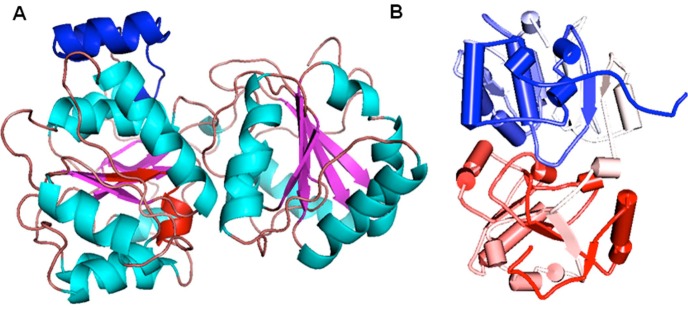
(A) Structural modelling of PgeIF4A2 showing ATPdependent RNA helicase DEAD-box conserved domain and RNA helicase DEAD-box type, Q-motif in red and blue respectively as visualized by Pymol. (B) Cartoon representation of PgeIF4A2 model showing its N- and C- termini in red and blue, respectively as visualized by Discovery Studio.

The PgeIF4A2 protein sequence comprises of 407 amino acid residues. The structural searching using PDB blast analysis revealed 77% sequence identity to PgeIF4A3 from H. sapiens (PDB ID : 2J0S) with an e-value of 0.0. ScanProsite server identified the fragment VLDEADEML as the ATP-dependent RNA Helicase DEAD-Box conserved site signature motif (181-189 residues) as a consensus pattern in both. Sequence alignment revealed that RNA helicase DEAD-box type, Q-motif was also 80% conserved in both target and template eIF4A sequences (Figure 1 & 2). Further, we developed three model structures for PgeIF4A2 using Modeller 9.12. Further refinement and validation was done with the model with the lowest Discrete Optimized Protein Energy, a statistical potential used to assess structural models) score of - 324317.04 as it was considered to be thermodynamically stable. It was latter visualized by PyMOL Molecular Graphics System.

### Validation of PgeIF4A2 structure

Modeller 9.12 was used for evaluation of the stereo-chemical quality and accuracy of the predicted model and this is based on Ramachandran plot calculation. Good stereo-chemical quality of the models is generally represented by a score which is close to 100 %. Torsion angles Ø and Ψ were used to examine the reliability of the PgeIF4A2. The protein structure was evaluated by a percentage quality measurement which included four kinds of occupancies: core, allowed, generously allowed and disallowed regions . The Modeller 9.12 generated model revealed 88.4 % residues falling in favoured region, 10.6 % residues in allowed region, and 1.0 % residues in outlier region while no residues were found in the disallowed region of the Ramachandran plot.

**Figure 3:**
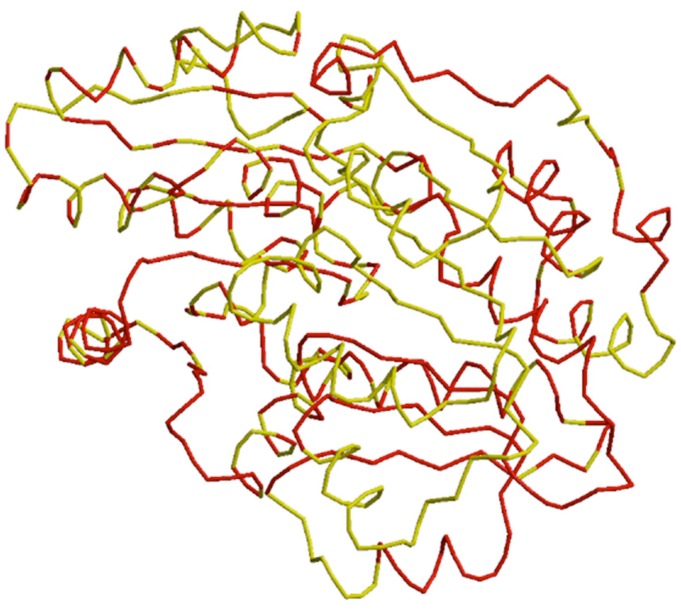
Superposition of Cα backbone of PgeIF4A2 (target) and HseIF4A (template) by SuperPose Version 1.0and visualized by Pymol.

ProSA-Web analysis of the model gives a Z-score value of the PgeIF4A2. The Z-score is indicative of the overall quality of PgeIF4A2 protein structure. Using it, the deviation of the total energy of the PgeIF4A2 is measured with respect to an energy distribution derived from random conformations. For a clear interpretation of the Z-score of the specified protein, its particular value is displayed in a plot which also contains the Z-score of all experimentally determined protein chains in PgeIF4A2 . Different colors (NMR in dark blue and X ray in light blue) are used to differentiate groups of structures from different sources (X-ray, NMR). Another application of this plot is to ascertain whether the Z-score of the PgeIF4A2 is within the range of scores typically found for proteins of similar size belonging to one of these groups. Z-score value (-9.66) of the PgeIF4A2 is located within the space of proteins determined by X ray crystallography and NMR. This value was extremely close to the value of template HseIF4A3 (-9.57), which inferred that the model thus obtained was reliable and very close to experimentally determined structures. Verify3D showed 94.87 % of the residues had a score greater than 0.2 that corresponded for a good quality model. RMSD was performed between equivalent atom pairs to measure the degree of structural similarity. RMSD analysis of the PgeIF4A2 model was measured from its HseIF4A3 (2J0S) from Homo sapiens using MOE software. The Cα RMSD and backbone RMSD deviation for the PgeIF4A2 model and the HseIF4A3 template crystal structure were 1.02 Å, and 1.03 Å respectively and over all RMSD was 1.61 Å (Figure 3). Thus, the PgeIF4A2 model generated by Modeller 9.12 was confirmed to be reliable and accurate.

### Comparative structural alignment of PgeIF4A2

**Figure 4:**
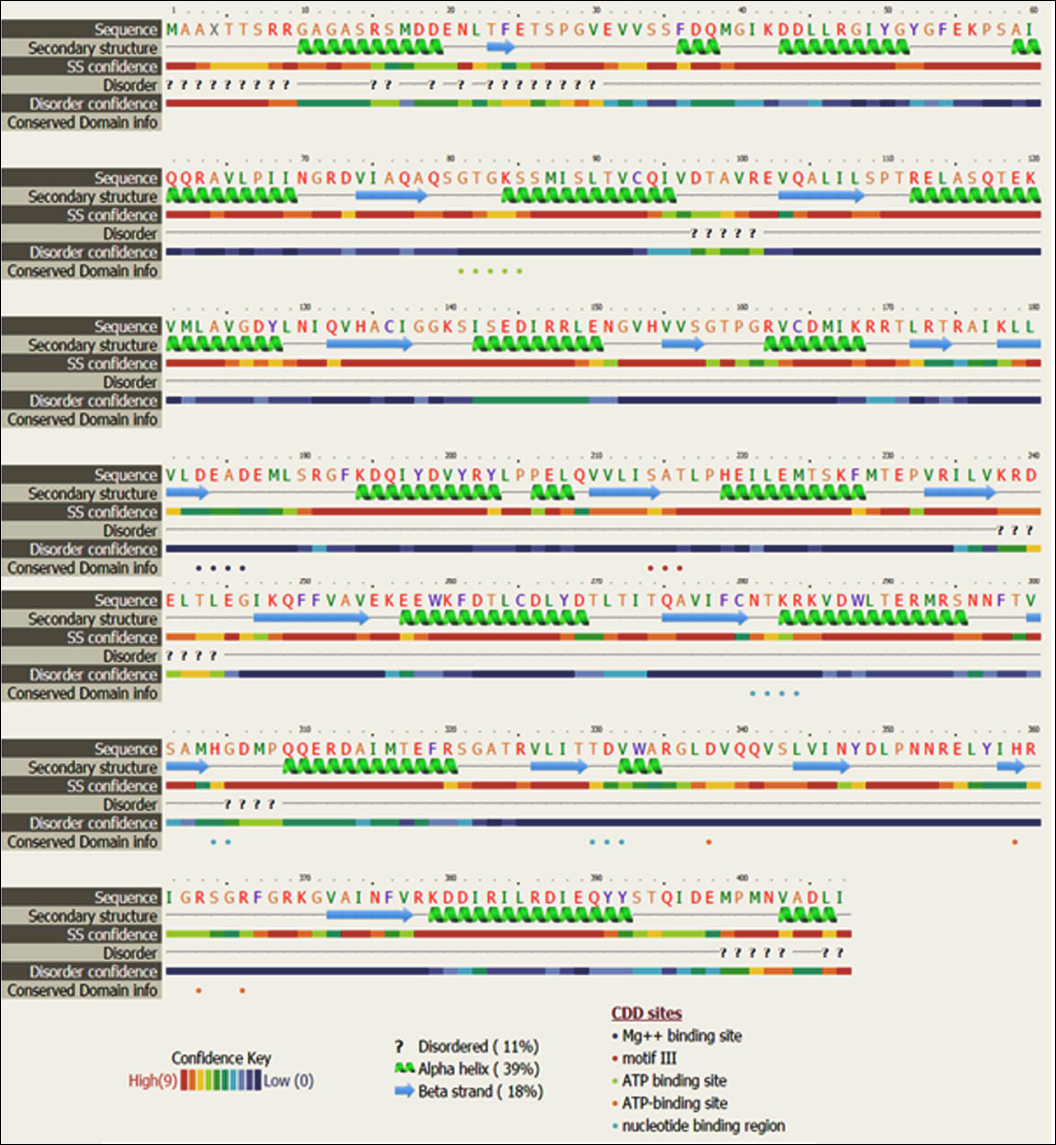
Comparative structure sequence alignment of deduced amino acid sequences of PgeIF4A2 along with other homologues.

Alignment of PgeIF4A2 was done with its homologous sequences and it revealed that 15 helices and 14 strands were conserved in all the selected species (Figure 4). High sequence similarity was observed in the entire sequence. The ATP-dependent RNA Helicase DEAD-box conserved site and RNA helicase DEAD-box type, Q-motif signature motifs were present in sheet-turn-helix and right handed alpha helix regions respectively (Figure 3). Our analysis revealed that the alpha helix might be involved in helicase activity. In addition, it revealed that the ATP-dependent RNA Helicase DEAD-box signature consensus pattern VLDEADEML) was conserved among all sequences. Thus, the model provides insight into the molecular function of the two conserved helicase domains in PgeIF4A2 in response to stress.

### Phylogenetic analysis

**Figure 5:**
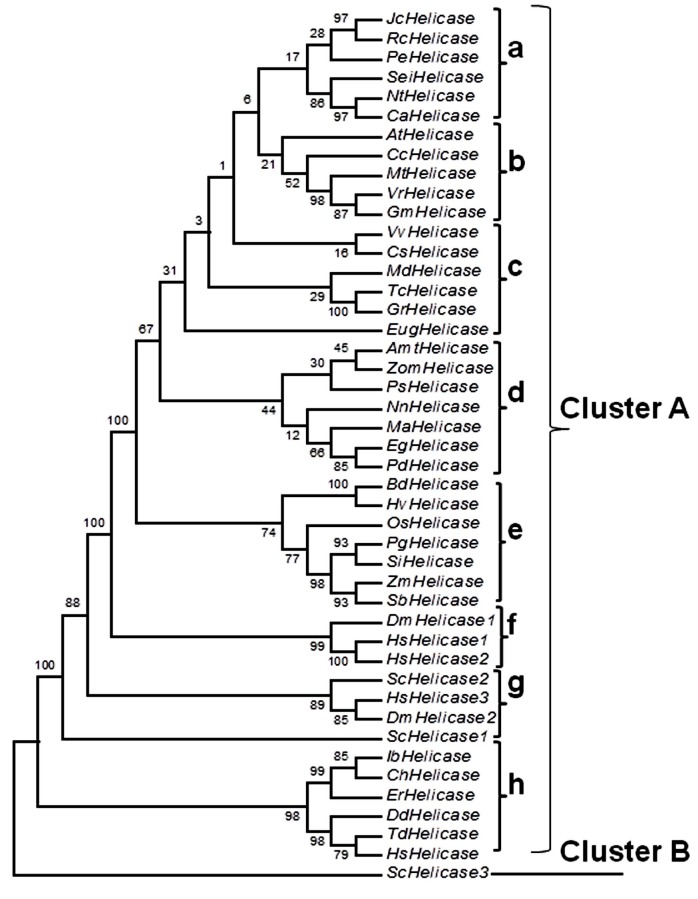
Phylogenetic analysis showing similarity of Pennisetum glaucum (PgeIF4A2) with other eIF4As from plants (dicots and monocots), mammals and microbes employing Molecular Evolutionary Genetics Analysis (MEGA) software version 4.0.

The RNA helicase family PgeIF4A2 is highly conserved among all higher plants, animals and prokaryotic organisms. It is a multi-gene family with each gene having multiple isoforms. Duplication and divergence led to the ubiquity of the PgeIF4A2 helicase family. To decipher the phylogenetic relationship of PgeIF4A2 with other plant, mammal and microbial orthologs, protein sequences available in the NCBI database were selected. eIF4As from seven monocots, twenty four dicots, and fourteen microbes and mammals were chosen for the phylogenetic analysis. The analysis (Figure 5) showed a clear demarcation of these eIF4As into two prominent clusters: cluster A and B. Cluster B comprised of only Saccharomyces cerevisiae and the rest were grouped in cluster A. Further, cluster A was heavily divided into sub groups. The subgroup a, b, c and d of cluster A included all the dicots while subgroup e had all the monocots including our target Pennisetum glaucum eIF4A. Subgroup f, g and h included all the microbial and mammal eIF4As. Therefore, our target eIF4A is closer phylogenetically to the subgroup including human helicases 1 and 2. However, PgeIF4A2 is closest to Oryza sativa and Setaria italica helicases. All the major clusters gave bootstrap values higher than 60. The tree showed distinct crop-specific clustering of sequences, revealing clear crop specific sequence differences.

## Conclusion

Eukaryotic translation initiation factors are a key component for the regulation of translational machinery and also play a significant role in providing stress tolerance to plants. Therefore, the model structure of PgeIF4A2 will be extremely crucial in establishing its molecular function in response to plant stress. To the best of our knowledge this is the first report regarding the structural modelling of Pennisetum glaucum PgeIF4A2. This model provides a perception into the molecular functions of ATPdependent RNA Helicase DEAD-box conserved site, helicase Cterminal and RNA helicase Q motif signature motifs during stress resistance in plants.
